# Antibody-Related Mechanisms in Ocular Diseases: Autoimmunity, Malignancy, and Therapeutic Targeting

**DOI:** 10.3390/antib15050094

**Published:** 2026-10-08

**Authors:** Yehan Xiao, Yaru Zou, Mingming Yang, Wendong Gu, Zizhen Ye, Jiaxin Deng, Kyoko Ohno-Matsui, Koju Kamoi

**Affiliations:** Department of Ophthalmology & Visual Science, Graduate School of Medical and Dental Sciences, Institute of Science Tokyo, Tokyo 113-8510, Japan; xyehan2002@gmail.com (Y.X.); alicezouyaru519@gmail.com (Y.Z.); yangmm-12@outlook.com (M.Y.); gwd981107@gmail.com (W.G.); yezizhen518@gmail.com (Z.Y.); dengjiaxin.med@gmail.com (J.D.); k.ohno.oph@tmd.ac.jp (K.O.-M.)

**Keywords:** autoantibodies, B cells, ocular autoimmunity, blood–ocular barrier, autoimmune retinopathy, optic neuritis, ocular myasthenia gravis, thyroid eye disease, ocular lymphoma, complement, monoclonal antibodies, targeted therapy

## Abstract

Antibodies in ocular disease have heterogeneous biological meanings: they may act as direct pathogenic effectors, systemic diagnostic biomarkers, indicators of B-cell clonality, or therapeutic agents. This distinction is particularly important in the eye, where immune privilege and the blood–ocular barrier restrict tissue access, whereas inflammation may permit entry of immunoglobulins, complement, and immune cells or support local humoral responses. This narrative review integrates antibody and B-cell biology across autoimmune and paraneoplastic retinopathies, aquaporin-4-immunoglobulin G-positive neuromyelitis optica spectrum disorder, myelin oligodendrocyte glycoprotein antibody-associated disease, ocular mucous membrane pemphigoid, ocular myasthenia gravis, thyroid eye disease, systemic autoimmune and fibroinflammatory disorders with ocular involvement, and B-cell malignancies including vitreoretinal and ocular adnexal lymphoma. We distinguish the evidence for direct antibody pathogenicity from disease association and emphasize that a positive result is interpretable only in relation to the ocular phenotype, assay platform, titer, specimen compartment, and orthogonal confirmation. Serum testing, ocular-fluid analysis, electrophysiology, multimodal imaging, cytology, histopathology, immunophenotyping, immunoglobulin clonality, and molecular testing have complementary roles. Therapeutic strategies include B-cell or B-lineage depletion, complement inhibition, cytokine- or receptor-directed monoclonal antibodies, insulin-like growth factor-1 receptor blockade, and emerging FcRn- or plasma-cell-directed approaches; however, regulatory status and ocular-specific evidence vary substantially among diseases. A mechanistically stratified framework may reduce overdiagnosis, improve recognition of malignant masquerade syndromes, and support more precise selection of targeted therapy.

## 1. Introduction

The eye is a highly specialized sensory organ in which uncontrolled inflammation may result in irreversible structural damage and permanent visual loss. To preserve visual function, the eye maintains a unique immune-regulatory environment known as ocular immune privilege [1]. Ocular immune privilege is maintained by several anatomical and functional mechanisms including the blood–ocular barrier (BOB), immunosuppressive factors in the aqueous humor, and an immunosuppressive intraocular microenvironment established in part by TGF-β-mediated immune regulation and anterior chamber-associated immune deviation [2]. Rather than eliminating immune responses entirely, ocular immune privilege maintains a controlled level of immunity that protects against pathogens while minimizing immune-mediated tissue damage [3]. Ocular-resident pigment epithelial cells also contribute actively to this immunoregulatory environment. Human iris pigment epithelial cells suppress bystander T-cell activation through TGF-β-TGF-β receptor signaling, illustrating a cellular mechanism that helps maintain ocular immune homeostasis [4].

A crucial part of the ocular immune privilege is the BOB, which includes the blood–retinal barrier (BRB) and the blood–aqueous barrier (BAB) [5]. They separate the anterior chamber, posterior chamber and retina from the bloodstream, preventing pathogens and toxins from entering the eye, controlling the flow of fluids, proteins and metabolites, and helping to maintain the immune status of the eyes [6].

However, ocular immune privilege can be disrupted by local or systemic inflammatory processes [7]. Inflammatory mediators may compromise the BOB, while barrier breakdown facilitates immune-cell infiltration and further amplifies inflammation, creating a self-sustaining cycle that may maintain disease activity [8]. Even after an initial infectious or inflammatory trigger has resolved, inflammation may persist and lead to progressive, irreversible tissue damage [9]. Importantly, BOB breakdown should be regarded as a permissive and relatively nonspecific event rather than as evidence of antibody-mediated pathology per se. Loss of barrier integrity can facilitate the entry of circulating immunoglobulins, complement components, and immune cells into ocular compartments, while local recruitment and activation of B cells may support intraocular humoral responses [10,11]. Conversely, BOB disruption may also arise from predominantly cellular or malignant infiltration. In HTLV-1-associated ocular disease, a rapid increase in circulating adult T-cell leukemia cells has been associated with the development of uveitis and disruption of the BOB [12,13]. This example illustrates that barrier dysfunction itself does not define the biological mechanism of ocular inflammation. Increased barrier permeability exposes ocular tissues to circulating immune components, whereas local recruitment, activation, and differentiation of B cells may promote autoantibody production. B cells also regulate T-cell activation and differentiation through antibody-independent functions [10]. By contrast, regulatory B cells (Bregs) exert immunosuppressive effects by inhibiting proinflammatory cytokine production and limiting excessive inflammatory responses [14]. Thus, the balance between pathogenic and regulatory B-cell functions plays an important role in the initiation, amplification, and resolution of antibody-related ocular inflammation.

Despite substantial advances in ocular immunology, an important interpretive gap remains. The presence of an antibody or a B-cell-associated marker does not have a uniform biological meaning across ocular diseases. Depending on the clinical context, an antibody or B-cell-associated finding may have different biological meanings: it may directly contribute to disease pathogenesis, serve primarily as a diagnostic or phenotypic biomarker, arise secondarily as a consequence of tissue injury, or indicate clonal B-cell proliferation. Moreover, detection of an antibody in serum or ocular fluid does not by itself establish causality, because its significance depends on the ocular phenotype, assay characteristics, antibody titer, specimen compartment, and evidence of local production or tissue injury [3,10,11]. Consequently, a framework that separates antibody pathogenicity from disease association, malignant B-cell clonality, and therapeutic antibody use is needed for clinically meaningful interpretation.

Accordingly, this review critically synthesizes antibody- and B-cell-associated mechanisms across ocular disease and distinguishes four biologically different roles: pathogenic antibodies, systemic diagnostic biomarkers, B-cell clonality or humoral biomarkers of malignancy, and therapeutic monoclonal antibodies. We integrate mechanistic evidence with specimen-specific diagnostic interpretation and the strength of ocular therapeutic evidence, with particular attention to situations in which antibody positivity does not establish causality.

## 2. Mechanistic Framework of Antibody-Mediated Ocular Injury

The mechanism of antibody-related ocular disease is complex. Under physiological conditions, ocular immune privilege prevents excessive immune activation. Under pathological conditions, this immune-privileged state may be disrupted, thereby contributing to ocular inflammation and tissue injury [15]. In this section, we discuss the major mechanisms of antibody-related ocular diseases, including BOB breakdown, autoantibody production, B-cell and plasma-cell activation, immune-complex formation, complement activation, and cytokine-mediated amplification of inflammation. These mechanisms often interact to sustain ocular inflammation and tissue injury across autoimmune diseases, infections, and malignant conditions [8,10].

### 2.1. Blood–Ocular Barrier Breakdown as a Permissive Mechanism

As mentioned earlier, the BOB is divided into two parts, namely the BRB and BAB [5].

The BRB consists of the inner and outer BRB. The inner BRB is established by the tight junctions between neighboring retinal endothelial cells and the outer BRB is formed by tight junctions between neighboring retinal pigment epithelial (RPE) cells. It plays an important role in the microenvironment of the retina and retinal neurons. This ensures that although the levels of amino acids or fatty acids in the blood fluctuate over a wide range, they do not affect the concentrations within the retina [16,17].

The BAB is established by tight junctions between the nonpigmented epithelial (NPE) cells of the ciliary epithelium [18]. Contemporary concepts suggest that the BAB should be regarded not only as a passive barrier between blood and aqueous humor, but also as a regulated interface for plasma protein entry into the anterior chamber [19].

When ocular inflammation occurs, BOB permeability may increase; disruption of the BRB in particular can contribute to macular edema and retinal fluid accumulation. Increased permeability allows circulating antibodies, complement components, and immune complexes to enter ocular tissues, thereby amplifying local inflammation [20].

An antibody detected in aqueous or vitreous fluid may reflect passive leakage across a disrupted barrier, local synthesis by recruited B cells or plasma cells, or both. Paired serum–ocular-fluid analysis, correction for total immunoglobulin and barrier permeability, and—in selected infectious or antigen-specific contexts—an antibody index or Goldmann–Witmer coefficient can help distinguish local production from nonspecific transudation. These approaches have methodological limitations and should be interpreted together with inflammation severity and sample timing [21].

### 2.2. Autoantibody Production

Autoantibodies represent an important component of humoral immune dysregulation in ocular disease. Ocular inflammation may promote BOB breakdown, impair immune tolerance, and allow immune cells to enter ocular tissues, where they may generate antibodies against ocular or systemic self-antigens [22].

Autoimmune retinopathy is a representative example. Circulating antiretinal antibodies may recognize retinal antigens and contribute to retinal degeneration, although whether these antibodies are primary drivers or secondary markers of tissue injury remains debated [23]. Cancer-associated retinopathy is a rare paraneoplastic visual syndrome defined as an autoantibody-driven remote effect of systemic cancer associated with retinal degeneration that is not caused by tumor metastasis [24].

Autoantibodies may not only initiate retinal degeneration by targeting retinal antigens, but also exacerbate disease progression when tissue injury exposes previously sequestered antigens and perpetuates the autoimmune response [25].

### 2.3. B-Cell and Plasma-Cell Activation

B-cell activation and differentiation provide the general immunological background for humoral immune responses, but these processes are not inherently specific to ocular disease. Following antigen recognition and appropriate T-cell help, activated B cells may undergo germinal-center reactions, class switching, and affinity maturation and subsequently differentiate into memory B cells, plasmablasts, or long-lived plasma cells [26]. The relevance of these processes to ocular pathology is disease-dependent and requires evidence of disease-specific autoantibodies, local B-cell or plasma-cell infiltration, intraocular antibody production, or other functional links to tissue injury.

Beyond antibody secretion, B cells may influence inflammation through antibody-independent functions, including antigen presentation, cytokine production, and modulation of T-cell responses [10,27]. In noninfectious ocular inflammation, B-cell participation has been reported in several disease settings, but the magnitude and functional importance of this contribution vary substantially among disorders [10,28]. Therefore, evidence of B-cell activation in blood or tissue should not by itself be interpreted as proof of direct B-cell-mediated ocular injury.

Disease-specific examples illustrate these distinctions. In Sjögren syndrome, B-cell hyperactivity and local lymphocytic infiltration are important components of disease biology, including involvement of the lacrimal gland; however, circulating anti-SSA/Ro or anti-SSB/La antibodies should not be assumed to directly mediate ocular-surface injury [29]. In IgG4-related ophthalmic disease, infiltration of IgG4-positive plasma cells is a characteristic histopathological feature involving the lacrimal gland, orbit, extraocular muscles, and other ocular adnexal tissues [30,31]. Nevertheless, the direct pathogenic role of IgG4 antibodies remains incompletely defined.

Thus, B-cell and plasma-cell biology provides an important mechanistic context for selected antibody-associated ocular diseases, but its significance should be established on a disease-specific basis rather than inferred from normal B-cell differentiation alone. Evidence is most convincing when supported by tissue localization, local antibody production, functional studies, or therapeutic responses.

In conclusion, B cells and plasma cells contribute to selected antibody-associated ocular diseases, linking humoral immunity to local inflammation [10,32].

### 2.4. Immune Complexes, Complement Activation, and Cytokine-Mediated Amplification

Immune complexes and cytokine-driven inflammation represent interconnected mechanisms that amplify antibody-related ocular disease. The complement system plays a crucial role in immunity, linking innate and adaptive immune responses and promoting antigen clearance. Once the complement system is activated, a chain reaction that involves proteolysis and assembly occurs, which results in cleavage of the third component of complement (C3). Activated C3 generates effector molecules that promote opsonization, generate inflammatory mediators, and recruit and activate leukocytes [33]. In Behçet syndrome (BS), C1q-high monocytes have been reported to accumulate and to exhibit enhanced phagocytic activity and secretion of inflammatory mediators, potentially contributing to disease progression [34].

Proinflammatory cytokines and chemokines have been shown to contribute to BOB breakdown in diabetic retinopathy [35]. In autoimmune ocular diseases, cytokines and chemokines such as TNF-α, IL-1β, IL-6, IL-8, IL-17, and CCL2 contribute to immune-cell recruitment, resident ocular-cell dysfunction, and BRB disruption [36].

Immune complexes, complement activation, and cytokine-mediated inflammation may reinforce one another. Immune complexes can activate the complement, complement fragments can recruit inflammatory cells, and cytokines produced by infiltrating immune cells may further disrupt barrier integrity.

Taken together, antibody-related ocular disease should not be viewed as the result of a single mechanism. It reflects the interaction of systemic immune dysregulation, BOB breakdown, B-cell activation, autoantibody, immune complex, complement activation, and cytokine-mediated inflammatory amplification. The relative contribution of each mechanism varies among diseases, providing a useful way to understand autoimmune and malignant ocular manifestations. Clinically, understanding these mechanisms may improve the interpretation of antibody and inflammatory biomarkers and help identify dominant pathogenic pathways in ocular diseases. This may support more rational selection of mechanism-based therapies.

## 3. Disease Spectrum and Biological Meaning of Antibodies

In this review, antibody- and B-cell-associated ocular disorders are organized into four clinically and mechanistically distinct categories: (1) ocular disorders with autoantibodies that have direct or probable pathogenic roles; (2) systemic autoimmune and fibroinflammatory diseases in which antibodies primarily serve as diagnostic or phenotypic biomarkers; (3) boundary disorders dominated by cellular or cytokine-mediated inflammation without an established disease-specific pathogenic ocular antibody; and (4) B-cell malignancies in which clonality, cytokine, and molecular markers support diagnosis. The representative diseases, ocular phenotypes, relevant antibody or B-cell markers, biological roles, and major interpretive caveats are summarized in Table 1.

### 3.1. Pathogenic Autoantibodies

#### 3.1.1. Autoimmune Retinopathy, Cancer-Associated Retinopathy, and Melanoma-Associated Retinopathy

Autoimmune retinopathy (AIR) comprises a heterogeneous group of rapidly progressive retinal degenerations characterized by visual symptoms, electrophysiological dysfunction, and circulating antiretinal antibodies. AIR may occur without an underlying malignancy or as a paraneoplastic disorder, principally cancer-associated retinopathy (CAR) and melanoma-associated retinopathy (MAR) [51]. In CAR, antibodies may arise through cross-reactivity between tumor antigens and retinal proteins, whereas MAR is classically associated with antibodies against bipolar-cell signaling components such as TRPM1 [37]. Recoverin is among the best-characterized antigens and can induce calcium-dependent photoreceptor apoptosis in experimental systems; however, many other antiretinal antibodies, including antibodies against α-enolase and carbonic anhydrase II, are less disease-specific [52,53]. Thus, the strength of evidence for causality differs substantially among individual antiretinal antibodies. They may act as pathogenic mediators, disease-associated biomarkers, or secondary products of retinal injury [53].

The central diagnostic challenge is that antiretinal antibodies may also be detected in healthy individuals, systemic autoimmune disease, cancer without retinopathy, and unrelated retinal degeneration. For example, in one cohort study, antiretinal antibodies were detected in 93% of patients without AIR, indicating their limited specificity [38]. Therefore, the diagnosis of AIR should not only rely on the serum antiretinal antibody, but also incorporate malignancy evaluation, electroretinogram (ERG), fluorescein angiogram, fundus autofluorescence, and optical coherence tomography (OCT) [54].

#### 3.1.2. AQP4-IgG-Positive Neuromyelitis Optica Spectrum Disorder and MOG Antibody-Associated Disease

Neuromyelitis optica spectrum disorder (NMOSD) is an autoimmune inflammatory disorder of the central nervous system that frequently involves the optic nerves and spinal cord. NMOSD-associated optic neuritis is often severe and may be bilateral or rapidly sequential, with posterior optic-nerve and chiasmal involvement being characteristic patterns [55]. Aquaporin-4 (AQP4) is widely expressed in the brain, spinal cord, and optic nerves. AQP4-IgG antibodies are predominantly of the IgG1 subclass. When AQP4-IgG binds to AQP4, it activates the classical complement pathway, disrupts the blood–brain barrier, and induces complement-mediated astrocytic injury, resulting in granulocyte infiltration, oligodendrocyte damage, and secondary demyelination. By contrast, MOG-IgG-associated disease (MOGAD) often presents with bilateral optic neuritis, marked optic disk edema, and recurrent attacks, although the phenotype varies with age [41]. Although human histopathological data remain limited, experimental evidence supports a pathogenic role for MOG-IgG. MOGAD patient-derived antibodies can mediate complement-dependent cytotoxicity, antibody-dependent cellular phagocytosis, and antibody-dependent cellular cytotoxicity, while experimental models indicate that both complement- and Fc receptor-dependent mechanisms contribute to MOG-IgG-mediated demyelination. Nevertheless, the overall pathogenesis of MOGAD remains incompletely understood [41,56,57].

Serum cell-based assays are the preferred method for detecting both antibodies. Serum AQP4-IgG testing is a key diagnostic test in patients with suspected NMOSD. However, a positive result alone is insufficient to establish the diagnosis; it should be interpreted together with a comprehensive physical and neurological examination and MRI findings [40]. Similarly, the detection of serum MOG-IgG is essential for diagnosing MOGAD. Nevertheless, false-positive results may occur. Therefore, MOG-IgG positivity should be interpreted in conjunction with compatible clinical manifestations and MRI findings to reduce the risk of misdiagnosis [58,59].

#### 3.1.3. Ocular Mucous Membrane Pemphigoid

Ocular mucous membrane pemphigoid (OMMP) is a chronic autoimmune cicatrizing conjunctivitis caused by immune responses against epithelial basement-membrane-zone proteins. In the early stage of OMMP, manifestations include dry eye with burning, itching, foreign-body sensation, and tearing, but these manifestations are nonspecific. During the development of OMMP, inflammatory-cell infiltration and fibroblast activation drive extracellular-matrix remodeling and progressive cicatricial changes, resulting in eyelid abnormalities such as lagophthalmos, entropion, lid-margin keratinization, and trichiasis [60].

OMMP is characterized by the linear deposition of IgG and IgA autoantibodies along the epithelial basement membrane zone (BMZ), where they target several structural proteins, including BP180, BP230, laminin-332, integrin α6β4 and type VII collagen [60,61]. Autoantibody binding, complement deposition, inflammatory-cell recruitment, and fibroblast activation contribute to progressive conjunctival scarring, symblepharon, limbal stem-cell failure, and visual loss. However, no single serum assay is sufficiently sensitive in all patients [62]. Although integrin β4 has been proposed as an important autoantigen in OMMP, its diagnostic utility requires further validation [63].

The diagnosis of OMMP, as an ocular manifestation of mucous membrane pemphigoid (MMP), is based on clinical findings together with detection of anti-BMZ autoantibodies. Demonstration of linear immunoglobulin and/or complement deposition along the BMZ by direct immunofluorescence (DIF) and biopsy is an important diagnostic criterion [64]. Nevertheless, DIF has limited sensitivity, and false-negative results have been reported in up to 50% of patients. Therefore, in patients with a compatible clinical phenotype but negative conjunctival DIF findings, biopsy of another mucosal site and additional serological testing may provide supportive evidence [65]. Thus, the diagnosis should not depend on a single serum antibody assay or biopsy result.

#### 3.1.4. Ocular Myasthenia Gravis

Ocular myasthenia gravis (OMG) is an antibody-mediated disorder of neuromuscular transmission that presents with fluctuating unilateral, bilateral, or alternating ptosis in combination with double vision. Ptosis usually becomes more pronounced toward the evening and improves with sleep or rest [66].

Acetylcholine receptor (AChR) antibodies are the dominant serological marker, whereas muscle-specific kinase (MuSK) and low-density lipoprotein receptor-related protein 4 (LRP4) antibodies are less frequent in purely ocular disease; consequently, seronegativity does not exclude the diagnosis [67]. AChR antibodies impair transmission through receptor blockade, accelerated receptor internalization, and complement-mediated postsynaptic injury, whereas MuSK antibodies are predominantly IgG4 and disrupt agrin–LRP4–MuSK signaling with limited complement activation [42]. Diagnosis should integrate fatigable weakness, the ice-pack test where appropriate, antibody testing, repetitive nerve stimulation, and especially single-fiber electromyography in seronegative cases. Antibody titer may add prognostic information but should not be used as an isolated measure of ocular disease activity [68].

#### 3.1.5. Thyroid Eye Disease

Thyroid eye disease (TED) is an autoimmune orbitopathy characterized by proptosis, with additional manifestations including lacrimation, photophobia, ocular pain, diplopia, and impaired color vision [69]. TSH-receptor (TSHR) autoantibodies bind to TSHR expressed on orbital fibroblasts, while functional crosstalk between TSHR and insulin-like growth factor-1 receptor (IGF-1R) amplifies downstream signaling. This interaction promotes the production of hydrophilic glycosaminoglycans and the differentiation of orbital fibroblasts into myofibroblasts and adipocytes. Collectively, these processes contribute to extraocular-muscle enlargement through edema, orbital-fat expansion through adipogenesis, and fibrosis of the orbital connective tissue [43].

There are two principal approaches to measuring TSHR antibodies: competitive TSH-binding inhibitory immunoglobulin (TBII) assays and functional thyroid-stimulating immunoglobulin (TSI) bioassays [70]. TBII or TSI supports the TED context and may correlate with activity or prognosis, but the antibody level alone does not define orbital severity or treatment response [71].

Given the critical role of IGF-1R in the pathogenesis of TED, monoclonal antibodies targeting IGF-1R have emerged as a promising therapeutic strategy [72]. Unlike the pathogenic autoantibodies involved in TED, teprotumumab is a therapeutic monoclonal antibody that binds to IGF-1R and inhibits its downstream signaling. Randomized clinical trials have demonstrated improvements in proptosis, diplopia, disease activity, and quality of life in patients with active, moderate-to-severe TED. The IGF-1R-directed mechanism, clinical efficacy, durability of response, and important safety considerations of teprotumumab have recently been comprehensively reviewed [73]. Nevertheless, further studies are required to evaluate the long-term durability of treatment responses, potential adverse effects, and efficacy in broader patient populations [74].

### 3.2. Systemic Autoimmune and Fibroinflammatory Diseases with Antibody-Associated Biomarkers

#### 3.2.1. ANCA-Associated Vasculitis

ANCA-associated vasculitis (AAV) represents one of the clearest examples of antibody-related systemic disease with ocular involvement. It includes three major subtypes: granulomatosis with polyangiitis (GPA), microscopic polyangiitis (MPA), and eosinophilic granulomatosis with polyangiitis (EGPA) [75]. Ocular involvement in AAV may affect both anterior and posterior ocular structures. Conjunctivitis/episcleritis and scleritis are among the most characteristic manifestations, particularly in GPA. Other manifestations include dacryocystitis, lacrimal duct obstruction, retro-orbital mass, peripheral ulcerative keratitis, retinal hemorrhage, retinal vasculitis, and uveitis [76].

AAV develops when genetic and environmental triggers break immune tolerance, leading to ANCA production. ANCAs activate primed neutrophils and monocytes, causing endothelial injury and small-vessel necrotizing inflammation. T cells, macrophages, BAFF-driven B-cell survival, and C5a-mediated complement amplification further sustain vascular damage [77].

High-quality antigen-specific assays for PR3-ANCA and MPO-ANCA support the diagnosis of AAV in patients with compatible ocular and systemic manifestations. However, negative PR3-ANCA and MPO-ANCA results do not exclude AAV, whereas a positive result contributes to the diagnostic work-up but is not diagnostic by itself. Therefore, the diagnosis should be established by integrating serological results with clinical, radiological, and histopathological findings [44].

#### 3.2.2. Sjögren’s Syndrome

Sjögren’s syndrome (SS) is a systemic autoimmune disease characterized by immune-mediated dysfunction of the lacrimal and salivary glands [78]. The predominant ocular manifestation is dry eye (keratoconjunctivitis sicca), which can cause photosensitivity, chronic irritation, corneal epithelial damage, and blepharitis [79]. The development of SS involves a series of complex immune processes characterized by B-cell hyperactivity and autoantibody production. Serological markers, particularly anti-SSA/Ro and anti-SSB/La antibodies, are important diagnostic features and reflect systemic B-cell hyperactivity in SS [80].

However, these circulating antibodies should not be assumed to directly mediate lacrimal-gland or ocular-surface injury. Persistent and progressive focal inflammatory-cell infiltration can lead to lacrimal-gland damage [81]. T cells predominate in mild lesions, whereas B cells predominate in advanced lesions and show increased activation and autoantibody production [82,83]. In addition, recent studies have also identified antibodies against aquaporins in SS. These antibodies may alter the distribution, localization, and function of these proteins and thereby contribute to abnormal glandular secretion [84].

Meanwhile, the ocular-surface damage in SS is not caused by autoantibodies alone; it also involves cytokine-mediated damage. These cytokine pathways include type I IFN, IL-12, IL-23, BAFF, IFN-γ, and IL-17 and are upregulated in SS, acting together with T cells, B cells, and autoantibodies to promote disease progression [85].

Anti-SSA/Ro and anti-SSB/La should therefore be presented primarily as systemic diagnostic and phenotypic biomarkers rather than as proven direct mediators of corneal or lacrimal injury. The ocular phenotype is produced by interacting epithelial, neural, T-cell, B-cell, interferon, and tear-film abnormalities. This distinction is important to avoid equating seropositivity with antibody-mediated ocular-surface damage [29].

#### 3.2.3. Systemic Lupus Erythematosus

Similar to SS, systemic lupus erythematosus (SLE) is characterized by systemic immune dysregulation and circulating autoantibody production [86]. Ocular involvement in SLE includes dry eye, episcleritis or scleritis, retinopathy, choroidopathy, and optic neuropathy [87].

The principal immunological biomarkers of SLE include antinuclear antibodies (ANA), anti-double-stranded DNA (anti-dsDNA) antibodies, and anti-Smith (anti-Sm) antibodies, low complement levels, and positive antiphospholipid (APL) antibodies [45]. Mechanistically, the pathogenesis of SLE involves loss of immune tolerance, B-cell hyperactivity, autoantibody production, immune-complex formation, complement activation, and dysregulated type I interferon signaling. At the cellular and molecular levels, overactive B cells produce autoantibodies and activate autoreactive T cells. These antibodies can form immune complexes that cause direct organ damage or activate complement and deposit within the vasculature. During this process, self-antigens exposed by dying cells may be further recognized by the immune system, thereby amplifying the immune response [88,89]. The cytokine most closely related to SLE is type I IFN, which is produced by plasmacytoid dendritic cells (pDCs). In SLE, immune complexes can stimulate pDCs to produce more type I IFN, thereby contributing to dysregulation of adaptive immunity [90].

Clinically, a positive ANA test is an entry criterion for the current EULAR/ACR classification of SLE [91]. However, different immunological markers may be associated with different ocular phenotypes. For example, anti-dsDNA antibodies and C3 levels have shown significant correlations with corneal sensation, superficial punctate keratopathy, and Schirmer I test results, suggesting an association with dry-eye manifestations [92]. Antiphospholipid antibodies have also been associated with retinal vasculitis [46]. Treatments for SLE may also cause ocular complications, such as corticosteroid-induced cataract or glaucoma and hydroxychloroquine retinopathy, which should be distinguished from immune-mediated ocular involvement [87]. Serological testing should therefore be interpreted in conjunction with ocular manifestations rather than as a stand-alone marker of ocular SLE.

#### 3.2.4. IgG4-Related Ophthalmic Disease

IgG4-related ophthalmic disease (IgG4-ROD) represents ocular and orbital involvement within the broader spectrum of systemic IgG4-related disease (IgG4-RD) [93]. It commonly affects the lacrimal glands, extraocular muscles, orbital nerves, and other ocular adnexal tissues [94].

Elevated serum total IgG and IgG4 levels are frequently observed in IgG4-ROD, although they are not present in all patients. At present, the disease is thought to be driven by autoimmune mechanisms, but the initiating triggers and the relative contribution of individual immune pathways remain incompletely defined [95]. The clinical response to rituximab suggests that CD20+ B cells may contribute to disease pathogenesis [96]. Increased expression of Th2-associated cytokines, including IL-4, IL-5, and IL-13, has also been reported, suggesting the involvement of Th2-mediated immune responses [30]. Although IgG4-positive plasma-cell infiltration is characteristic of IgG4-ROD, the direct pathogenic role of IgG4 antibodies remains unclear.

Elevated serum IgG4 is an important serological feature of IgG4-ROD; however, it lacks sufficient specificity to establish the diagnosis when interpreted in isolation. Accurate diagnosis therefore requires an integrated assessment of the clinical phenotype, imaging findings, histopathological features, and other relevant laboratory results [31].

### 3.3. Boundary Disorders: Predominantly Cellular or Cytokine-Mediated Ocular Inflammation

The following disorders are included as boundary comparators because they illustrate ocular inflammation in which antibody-related findings have been reported, but no disease-specific pathogenic ocular autoantibody has been established. Their inclusion helps distinguish antibody-mediated disease from disorders in which cellular immunity, cytokine networks, granulomatous inflammation, or other immune pathways predominate.

#### 3.3.1. Behçet’s Syndrome

Behçet syndrome (BS) is a systemic inflammatory vasculitis with a complex and incompletely understood pathogenesis involving both innate and adaptive immune mechanisms [97]. Ocular involvement most commonly manifests as relapsing nongranulomatous uveitis with retinal vasculitis and may present as anterior, posterior, or panuveitis [47,97]. Unlike diseases defined by established pathogenic autoantibodies, no disease-specific autoantibody has been validated for the diagnosis or pathogenesis of BS. Immune responses against heat-shock proteins and other candidate antigens have been described, but their specificity and causal significance remain uncertain [98].

The etiology of BS remains incompletely understood. Genetic susceptibility and environmental triggers are thought to interact in promoting dysregulated innate and adaptive immune responses. Molecular mimicry has been proposed as one mechanism, whereby microbial antigens may cross-react with host proteins, including heat-shock proteins, tropomyosin, S-antigen, and interphotoreceptor retinoid-binding protein (IRBP). Such responses may contribute to ocular inflammation, although their direct causal role has not been established [47].

Ocular inflammation in BS is associated with dysregulated cytokine networks involving TNF-α, IFN-γ, IL-6, IL-17, and other mediators that promote neutrophil activation and Th1/Th17-skewed immune responses [47,99]. In contrast, interleukin-1 receptor antagonist (IL-1Ra) is an endogenous anti-inflammatory mediator that counterbalances IL-1 signaling and should not be classified as a proinflammatory cytokine. The clinical efficacy of TNF-targeted therapy and emerging evidence for other cytokine-directed approaches further support the importance of cytokine-mediated inflammation in Behçet uveitis [99,100,101].

The diagnosis of BS remains clinical and is not based on a disease-specific autoantibody [102]. Anti-tubulin-α-1c antibodies have been investigated as potential biomarkers, but current evidence is insufficient to establish their diagnostic utility or pathogenic relevance [103].

#### 3.3.2. Sarcoidosis

Sarcoidosis is a multisystem granulomatous inflammatory disease that can involve virtually any ocular structure. Uveitis is the most common ocular manifestation and may present as anterior, intermediate, posterior, or panuveitis; conjunctival and other adnexal manifestations may also occur [104]. Ocular disease can cause substantial visual morbidity, particularly when complicated by macular edema, epiretinal membrane, glaucoma, or other structural complications [105].

In contrast to diseases defined by pathogenic or disease-associated autoantibodies, sarcoidosis has no established disease-specific pathogenic antibody. Its inclusion here therefore serves as a boundary example of an immune-mediated ocular disease in which granulomatous cellular inflammation, rather than antibody-mediated injury, predominates.

Sarcoidosis is not classically regarded as an antibody-related disease; however, systemic immune biomarkers may support diagnosis and help distinguish sarcoid uveitis from other causes of ocular inflammation [48]. Serum angiotensin-converting enzyme has traditionally been used as a supportive biomarker, while other systemic immune markers, including lymphopenia and serum soluble IL-2 receptor (sIL-2R), may also support diagnosis [106,107].

Sarcoidosis may therefore be regarded as an immune-mediated ocular disease in which antibody-related mechanisms remain unclear, whereas systemic immune biomarkers can support diagnosis.

### 3.4. B-Cell Malignancies

Malignant lymphoma represents a distinct context in which B-cell lineage, intraocular cytokine profiles, and immunoglobulin clonality are diagnostically important. Hematologic malignancies may involve nearly all ocular compartments and can mimic inflammatory or infectious eye disease. Their evaluation therefore requires integration of ocular phenotype, imaging, ocular-fluid or tissue analysis, and systemic hematologic assessment [108]. Ocular involvement mainly includes vitreoretinal lymphoma and ocular adnexal lymphoma. Because many ocular lymphomas originate from B cells, malignant B-cell proliferation and associated humoral or cytokine biomarkers are clinically relevant [109,110]. In addition, patients with systemic autoimmune diseases such as SS have an increased risk of B-cell lymphoma [111]. Therefore, in this section, the molecular mechanisms of ocular lymphoma are also discussed.

#### 3.4.1. Vitreoretinal Lymphoma

Vitreoretinal lymphoma (VRL) is most commonly a diffuse large B-cell lymphoma and is closely related to primary central nervous system lymphoma [112]. The clinical manifestations of VRL include blurred or reduced vision and floaters. Typical findings include vitreous cell aggregates and multifocal cream-colored subretinal lesions, occasionally accompanied by retinal pigment epithelial atrophy, vascular occlusion, or optic-nerve involvement. VRL is a masquerade syndrome that often mimics chronic uveitis, making diagnosis challenging [113].

Immunological and molecular analyses of intraocular fluid can provide valuable supportive evidence for VRL. Elevated intraocular IL-10 levels and an increased IL-10/IL-6 ratio favor VRL over inflammatory uveitis. PCR-based detection of clonal immunoglobulin heavy-chain (IgH) gene rearrangements may further improve diagnostic sensitivity by demonstrating a monoclonal B-cell population [114]. Collectively, these findings reflect the malignant clonal B-cell proliferation characteristic of VRL [49].

Although the IL-10/IL-6 ratio, IgH gene-rearrangement analysis, and flow-cytometric detection of clonal B cells have demonstrated diagnostic utility, each method has important limitations. For example, infection-associated or reactive clonal B-cell expansion may produce false-positive IgH rearrangement results [115]. Therefore, VRL should not be diagnosed or excluded on the basis of any single test. Accurate diagnosis requires integrated interpretation of the clinical phenotype and multimodal imaging together with cytological, flow-cytometric, cytokine, and molecular findings. The complementary value of ocular-fluid testing is also illustrated by a case of myeloid/NK-cell precursor acute leukemia in which aqueous-humor flow cytometry identified malignant cells, while PCR contributed to the exclusion of infectious mimickers [116].

#### 3.4.2. Ocular Adnexal Lymphoma

Ocular adnexal lymphoma (OAL) comprises lymphomas involving the conjunctiva, orbit, lacrimal gland, eyelid, or lacrimal drainage system. Its clinical manifestations vary according to the anatomical site and may include a painless salmon-colored conjunctival mass, proptosis, eyelid swelling or ptosis, diplopia, restricted ocular motility, and lacrimal-gland enlargement [117].

Most OALs are B-cell lymphomas, and the detection of a monoclonal B-cell population can provide evidence of malignant B-cell proliferation [50]. Molecular and cytogenetic studies suggest that immune dysregulation and chronic antigenic stimulation may contribute to the pathogenesis of OAL [118]. Immunophenotyping of biopsy specimens by flow cytometry can support the diagnosis by demonstrating B-cell lineage and clonality [50]. Because individual immunophenotypic markers are not entirely specific, no single marker should serve as the sole basis for diagnosis [119].

## 4. Diagnosis and Therapeutic Targeting

Antibody-related mechanisms are clinically important not only for understanding disease pathogenesis, but also for guiding diagnosis and therapeutic decision-making. In systemic autoimmune diseases, autoantibodies may help identify the underlying disorder when ocular manifestations are the first or dominant clinical sign [120]. In malignant diseases, intraocular cytokine profiles and B-cell clonality or molecular markers may help distinguish lymphoma from uveitis [49]. From a therapeutic perspective, autoantibodies, B cells, complement activation, and cytokine pathways may serve as targets for mechanism-based therapy.

### 4.1. The Significance of Antibody-Related Biomarkers in Diagnosis

Antibody-related biomarkers have important diagnostic value in ocular diseases because they can help link ocular manifestations to systemic autoimmune disease or lymphoma.

**Figure 1:**
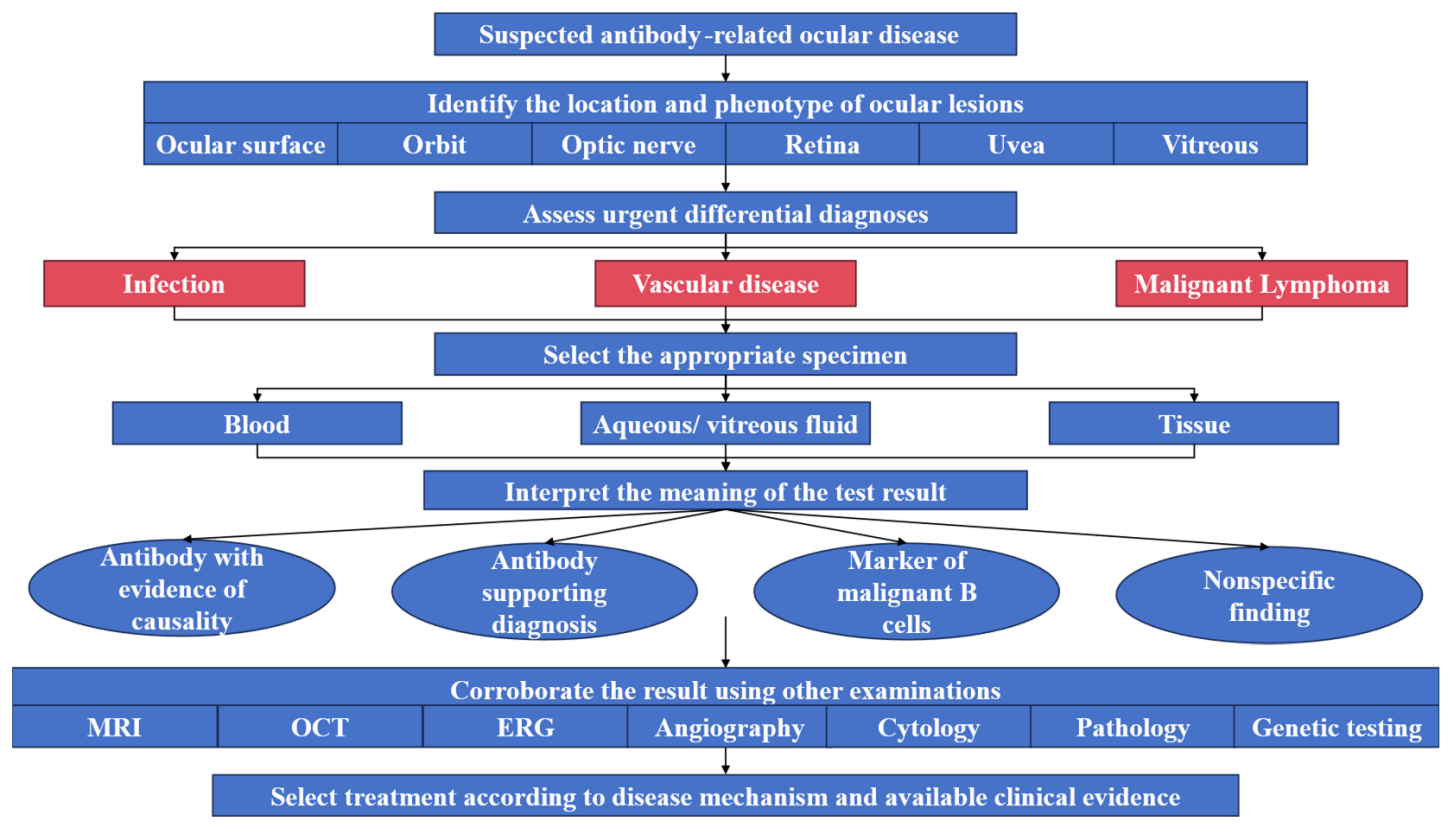
Diagnostic and therapeutic workflow for antibody-related ocular diseases.

Because many ocular manifestations of systemic immune-mediated diseases are nonspecific, systemic serological testing can provide important supportive evidence for identifying the underlying disorder (Table 2 and Figure 1). For example, serological testing may reveal anti-SSA/Ro and anti-SSB/La antibodies, rheumatoid factor, and polyclonal hypergammaglobulinemia as evidence of increased B-cell activity, which may support the diagnosis of SS [121]. A cohort study in SLE found that anti-dsDNA and anti-β2 glycoprotein I antibodies, positive Coombs tests, autoimmune hemolytic anemia, hypocomplementemia, and leukopenia may support systemic diagnosis and disease assessment. However, these findings are not specific to ocular involvement and should be interpreted in the context of the ocular phenotype [122]. Similarly, PR3-ANCA and MPO-ANCA are specific serological markers that support the diagnosis of AAV in an appropriate clinical context [77]. For IgG4-ROD, the presence of dense inflammatory cells within the tissue and the ratio of IgG4-positive cells to IgG-positive cells are essential conditions for diagnosis [31].

Immunological tests can also help distinguish uveitis from lymphoma. In VRL, elevated intraocular IL-10 levels and an increased IL-10/IL-6 ratio provide important supportive evidence for the diagnosis [123]. Furthermore, newer assays, such as immunoglobulin kappa light-chain clonality testing, may improve the diagnostic sensitivity for VRL [124]. However, these biomarkers must be interpreted cautiously. For example, in cases where the infiltration of cancer cells is severe, the ratio of IL-10 to IL-6 may not be typical [124]. Therefore, serological testing and intraocular fluid analysis should be integrated with ocular imaging, systemic evaluation, and histopathology when necessary.

**Table 2:** Diagnostic tests, preferred specimens, clinical use, and major limitations.

Marker/Test	Preferred Specimen/Assay	Main Clinical Use	Key Limitation/Interpretation	Key References
**PR3-ANCA/MPO-ANCA**	Serum; antigen-specific immunoassay	Supports AAV subtype and systemic diagnosis	Negative result does not exclude limited disease; titer alone does not define ocular activity	[44,77]
**Anti-SSA/Ro/anti-SSB/La**	Serum immunoassay	Supports Sjögren/SLE diagnosis and phenotype	Does not establish direct antibody-mediated ocular-surface injury	[29,80]
**Anti-dsDNA/complement**	Serum	Supports SLE activity and immune-complex biology	Relationship to a specific ocular lesion is indirect	[45,92]
**Antiphospholipid antibodies**	Serum; persistent positivity required clinically	Supports thrombotic mechanism in vaso-occlusive retinopathy	Transient positivity and incomplete criteria can mislead	[46]
**AQP4-IgG**	Serum; validated cell-based assay	Disease-defining biomarker in compatible NMOSD	Phenotype/MRI correlation required	[39,40]
**MOG-IgG**	Serum; cell-based assay with reported titer	Supports MOGAD in compatible optic neuritis	Low-positive results have lower positive predictive value	[58]
**Antiretinal antibodies**	Serum; laboratory-specific immunoblot/IHC	Support selected AIR/CAR/MAR phenotypes	Poor standardization; antibodies may occur without AIR	[38,54]
**Direct immunofluorescence**	Perilesional conjunctival biopsy	Supports ocular mucous membrane pemphigoid	Limited sensitivity; negative test does not exclude disease	[64,65]
**Serum IgG4/tissue IgG4+ cells**	Serum plus biopsy with histology/IHC	Support IgG4-ROD diagnosis	Serum elevation is nonspecific; histologic context is essential	[31,94]
**IL-10 and IL-10/IL-6 ratio**	Vitreous or aqueous	Supports VRL over inflammatory uveitis	Not definitive; thresholds and inflammatory context vary	[123]
**MYD88 L265P**	Ocular fluid/tissue; PCR or sequencing	Molecular support for VRL	Not present in all cases and not entirely disease-exclusive	[114,125]
**IgH/IgK clonality**	Ocular fluid or tissue	Supports monoclonal B-cell population	Pseudoclonality may occur in low-cellularity samples	[124]
**AChR/MuSK/LRP4** **antibodies**	Serum; validated immunoassay or cell-based assay	Supports autoimmune myasthenia in compatible fluctuating ptosis/diplopia	Seronegativity is more frequent in ocular-only disease; correlate with examination and electrophysiology	[42]
**TSH-receptor antibodies/** **thyroid-stimulating** **immunoglobulin**	Serum; platform-specific assay	Supports Graves’ disease/TED context and may inform activity or prognosis	Antibody level alone does not determine orbital severity or treatment response	[70,71]

Abbreviations: PR3, proteinase 3; ANCA, antineutrophil cytoplasmic antibody; MPO, myeloperoxidase; AAV, ANCA-associated vasculitis; SSA/Ro, Sjögren’s syndrome-related antigen A/Ro; SSB/La, Sjögren’s syndrome-related antigen B/La; SLE, systemic lupus erythematosus; dsDNA, double-stranded DNA; AQP4, aquaporin-4; IgG, immunoglobulin G; NMOSD, neuromyelitis optica spectrum disorder; MRI, magnetic resonance imaging; MOG, myelin oligodendrocyte glycoprotein; MOGAD, MOG antibody-associated disease; IHC, immunohistochemistry; AIR, autoimmune retinopathy; CAR, cancer-associated retinopathy; MAR, melanoma-associated retinopathy; IgG4-ROD, IgG4-related ophthalmic disease; VRL, vitreoretinal lymphoma; PCR, polymerase chain reaction; IgH, immunoglobulin heavy chain; IgK, immunoglobulin kappa light chain; AChR, acetylcholine receptor; MuSK, muscle-specific kinase; LRP4, low-density lipoprotein receptor-related protein 4; TED, thyroid eye disease.

### 4.2. Therapeutic Targeting of Antibody-Related Mechanisms

The therapeutic significance of antibody-related mechanisms is their ability to identify targetable immune pathways. Although not all ocular diseases discussed in this review are directly caused by pathogenic autoantibodies, many of them involve humoral immune activation, cytokine-mediated amplification, B-cell dysfunction, or malignant B-cell proliferation. Therefore, cytokine inhibition, B-cell depletion, complement modulation, or lymphoma-directed therapy has shown therapeutic benefit in the treatment of ocular disease (Table 3).

Cytokine-targeted therapy is particularly relevant in inflammatory ocular diseases in which autoantibodies are not the primary pathogenic drivers. BS is a representative example. As mentioned earlier, TNF-α, IFN-γ, IL-1, and IL-6 play important roles in BS [99]. Among these mediators, IL-6 contributes to immune activation, vascular permeability, and tissue injury, making IL-6 or IL-6 receptor blockade a potential option in selected refractory ocular inflammatory diseases. TNF inhibition has the strongest established biologic evidence for Behçet uveitis. Infliximab was more effective than ciclosporin in controlling ocular inflammation during the initial 6-month treatment period [126]. Long-term studies subsequently demonstrated sustained efficacy over 10 years, both in multicenter cohorts and in patients receiving infliximab monotherapy [127,128]. Nevertheless, ocular inflammation may recur after infliximab discontinuation or during maintenance treatment, underscoring the need for individualized long-term management [129,130,131]. Adalimumab has also demonstrated efficacy in noninfectious intermediate, posterior, and panuveitis [132,133]. In the VISUAL I and VISUAL II trials, adalimumab reduced the risk of treatment failure in patients with active or inactive noninfectious uveitis [133]. Japanese multicenter real-world data have also characterized the use of local and systemic therapies for recurrent noninfectious uveitis [134]. In refractory noninfectious uveitic macular edema, sub-Tenon’s triamcinolone used as bridging therapy to adalimumab was associated with earlier anatomical improvement than adalimumab monotherapy [135]. Tocilizumab, as an IL-6 receptor inhibitor, has also shown efficacy in the treatment of certain patients with uveitis [101].

B-cell-directed therapy provides a direct therapeutic link to humoral immune dysregulation. Rituximab is a chimeric anti-CD20 monoclonal antibody that targets CD20+ B cells [136]. A multicenter prospective phase II study evaluating the R2 regimen (rituximab, 375 mg/m^2^ intravenously on day 1; lenalidomide orally on days 1–21, 20 mg/day during cycle 1 and 25 mg/day thereafter in the absence of hematological toxicity) suggested that this regimen may be effective in selected patients when administered for eight 28-day treatment cycles [137]. Rituximab is also used in other B-cell-mediated diseases. For instance, in SS, rituximab has shown potential benefit in selected patients, although responses vary across studies [138]. In AAV, rituximab is an established B-cell-depleting therapy used for remission induction and maintenance in appropriate patients [139]. Rituximab has shown clinical benefit in selected patients with IgG4-ROD, although evidence remains limited [94]. Complement-targeted therapy also represents an important mechanism-based approach in ocular disease. In geographic atrophy secondary to age-related macular degeneration (AMD), intravitreal pegcetacoplan targets complement component C3, whereas avacincaptad pegol inhibits C5. Pivotal phase 3 trials have demonstrated that both agents can slow geographic atrophy lesion growth. However, further studies are needed to clarify their long-term efficacy, safety, and impact on visual outcomes [140,141,142].

Biologic therapy may reactivate or exacerbate latent or chronic infection, and this risk depends on the targeted immune pathway, host immune status, and concomitant immunosuppression [143]. A reported patient with HTLV-1 infection experienced exacerbation of HTLV-1-associated conditions during biologic treatment for rheumatoid arthritis, highlighting the need for infection-specific risk assessment [144]. Opportunistic viral retinitis, including cytomegalovirus retinitis, should also be considered in heavily immunosuppressed patients who develop new visual symptoms [145].

Conversely, context-specific preclinical studies using ocular HTLV-1 infection models did not demonstrate marked enhancement of viral burden or ocular inflammatory responses following exposure to infliximab or adalimumab [146,147]. Similar in vitro approaches have been used to evaluate tocilizumab and intraocular anti-VEGF therapy under HTLV-1-infected conditions [148,149]. These findings are reassuring but should not be interpreted as definitive evidence of clinical safety.

Overall, antibody-related and immune biomarkers can guide treatment. These targeted approaches may improve disease control in selected patients, but treatment decisions should be individualized according to disease type, severity, systemic involvement, and potential adverse effects.

**Table 3:** Mechanism-based therapeutic targets and level of ocular evidence.

Target/Representative Agent	Mechanistic Rationale	Relevant Ocular Disorder(s)	Evidence/Status to State	Major Caution	Key References
**CD20/rituximab**	B-cell depletion and reduced antigen presentation/autoantibody renewal	AAV, IgG4-ROD, selected OMMP; lymphoma	Established systemic use; ocular evidence varies by disease	Infection, hypogammaglobulinemia, HBV reactivation	[65,94,136,139]
**CD19/inebilizumab**	Broader B-lineage depletion including plasmablasts	AQP4-IgG NMOSD	Approved disease-modifying therapy in relevant jurisdictions	Infection and immunoglobulin reduction	[150]
**BAFF/belimumab**	Reduces survival of autoreactive B cells	SLE; possible ocular relevance through systemic control	Do not overstate direct ocular trial evidence	Delayed effect; infection	[151]
**C5/eculizumab or ravulizumab**	Prevents terminal complement-mediated injury	AQP4-IgG NMOSD; generalized AChR-positive myasthenia gravis with ocular manifestations	High-level systemic evidence; do not extrapolate generalized-MG trials to isolated ocular myasthenia	Meningococcal infection risk; vaccination required	[152,153,154]
**C3/pegcetacoplan**	Inhibits C3 activation	Geographic atrophy secondary to AMD	Reduces GA lesion growth	Intraocular inflammation and exudative AMD	[140,141]
**C5/avacincaptad pegol**	Inhibits C5 cleavage and downstream complement activation	Geographic atrophy secondary to AMD	Reduces GA lesion growth	Increased CNV and injection-related adverse events	[142]
**IGF-1R/teprotumumab**	Targets orbital fibroblast signaling	Active thyroid eye disease	Approved targeted antibody in relevant jurisdictions	Hearing, glucose and pregnancy-related precautions	[73]
**Intravitreal** **anti-CD20/rituximab**	Local treatment of malignant B cells	Selected VRL	Case series/selected protocols; systemic/CNS strategy remains essential	Ocular toxicity, recurrence, incomplete CNS control	[155]
**FcRn inhibition/** **efgartigimod, rozanolixizumab, and emerging agents**	Accelerates clearance of pathogenic IgG	Generalized myasthenia gravis; potential relevance to ocular symptoms and other IgG-mediated ocular disease	Approved for selected generalized-MG populations in relevant jurisdictions; isolated ocular-MG evidence remains limited	Infection and reduced IgG; avoid extrapolating generalized-MG efficacy to isolated ocular disease	[156]

Abbreviations: CD, cluster of differentiation; AAV, ANCA-associated vasculitis; IgG4-ROD, IgG4-related ophthalmic disease; OMMP, ocular mucous membrane pemphigoid; SLE, systemic lupus erythematosus; HBV, hepatitis B virus; AQP4, aquaporin-4; IgG, immunoglobulin G; NMOSD, neuromyelitis optica spectrum disorder; BAFF, B-cell activating factor; C5, complement component 5; AChR, acetylcholine receptor; C3, complement component 3; GA, geographic atrophy; CNV, choroidal neovascularization; IGF-1R, insulin-like growth factor-1 receptor; VRL, vitreoretinal lymphoma; CNS, central nervous system; FcRn, neonatal Fc receptor; MG, myasthenia gravis; AMD, age-related macular degeneration.

### 4.3. Integrated Interpretation and Limitations

Although antibody-related and immune biomarkers provide important diagnostic and therapeutic information, they should not be interpreted in isolation. A positive autoantibody test may support systemic immune dysregulation, but it does not establish direct antibody-mediated ocular injury [53].

An integrated diagnostic approach is therefore essential (Figure 1). Serological testing provides important diagnostic information, but accurate diagnosis requires integration with the patient’s medical history, symptoms, and imaging findings. During treatment, regular monitoring is necessary to identify treatment-related toxicity and prevent further ocular damage.

Treatment-related limitations also warrant attention. Therapeutic monoclonal antibodies may themselves disturb ocular immune homeostasis. Immune checkpoint inhibitors can induce a broad spectrum of ophthalmic immune-related adverse events, including uveitis, retinopathy, optic neuritis, orbital inflammation, ocular myopathy, and ocular-surface disease [157].

Antibody results should be interpreted in conjunction with the ocular phenotype, assay validity, specimen compartment, and confirmatory evidence. Abbreviations: MRI, magnetic resonance imaging; OCT, optical coherence tomography; ERG, electroretinography.

## 5. Knowledge Gaps and Future Directions

Future research should move beyond binary antibody positivity toward quantitative and functional phenotyping [158]. Priorities include standardized multicenter assays, epitope-level profiling, antibody subclass and Fc-glycosylation analysis, paired serum–ocular-fluid studies, and longitudinal sampling before and after targeted therapy. Studies using harmonized protocols should subsequently validate diagnostic sensitivity, specificity, and predictive value in independent cohorts [159]. For example, in NMOSD and MOGAD, cell-based assays, defined serological criteria, and integration with clinical and imaging findings improve diagnostic interpretation [59,160].

A second priority is the development of clinically useful causality frameworks [25]. Diagnostic studies should combine antibody results with objective ocular phenotypes, predefined assay thresholds, independent validation cohorts, and treatment-response data [38,54,161]. For rare disorders such as AIR and VRL, international registries and harmonized biobanking will be essential. Finally, trials should distinguish systemic disease control from direct improvement in ocular outcomes and should report vision, imaging, relapse, steroid-sparing effects, and treatment-related toxicity separately.

## 6. Conclusions

Antibodies in ocular disease may act as pathogenic effectors, systemic diagnostic biomarkers, indicators of malignant B-cell clonality, or therapeutic agents, and these roles are not interchangeable. Interpretation requires concordance among ocular phenotype, assay validity and titer, serum or ocular-fluid compartment, tissue or molecular confirmation, and the strength of evidence for causality. Applying this mechanistic framework may reduce overdiagnosis from isolated antibody positivity, improve recognition of malignant masquerade syndromes, and support selection of therapies directed at B cells, complement, Fc-dependent pathways, cytokine receptors, or disease-specific targets. Future progress will depend on standardized assays, paired serum–ocular-fluid studies, functional antibody characterization, and prospective trials with ocular endpoints.

## Figures and Tables

**Table 1:** Ocular phenotypes and biological roles of antibody- and B-cell-associated findings in representative ocular diseases.

Disease Category	Representative Disease	Key Ocular Phenotype	Antibody or B-Cell Marker	Role	Major Caveat	Key References
**Pathogenic** **autoantibody-associated ocular disorders**	Autoimmune retinopathy/CAR/MAR	Photopsia, field loss, outer-retinal dysfunction	Recoverin, α-enolase, TRPM1 and others	Pathogenic or probably pathogenic in selected settings	Assay standardization and false-positive reactivity remain major limitations	[37,38]
	AQP4-IgG NMOSD	Severe optic neuritis, chiasmal/posterior involvement	AQP4-IgG	Disease-defining and pathogenic	Use validated cell-based assay; phenotype and MRI remain essential	[39,40]
	MOG antibody-associated disease	Optic disk edema, recurrent/bilateral optic neuritis	MOG-IgG	Disease-associated, likely pathogenic	Low-titer results require cautious clinical correlation	[41]
	Ocular myasthenia gravis	Fluctuating ptosis and diplopia; fatigable ocular weakness	AChR, MuSK, or LRP4 antibodies	Pathogenic neuromuscular-junction autoantibodies	Seronegativity does not exclude ocular-only disease; correlate with examination and electrophysiology	[42]
	Thyroid eye disease	Proptosis, diplopia, optic neuropathy	TSH-receptor antibodies	Disease-associated/pathogenic receptor antibody	Activity and severity are not determined by antibody level alone	[43]
**Systemic** **autoimmune and** **fibroinflammatory diseases with antibody-associated biomarkers**	ANCA-associated vasculitis	Scleritis, PUK, orbital disease, retinal vasculitis	PR3-ANCA or MPO-ANCA	Pathogenic systemic autoantibody with ocular vascular effects	Negative ANCA does not exclude limited GPA	[44]
	Sjögren syndrome	Keratoconjunctivitis sicca, lacrimal dysfunction	Anti-SSA/Ro, anti-SSB/La; B-cell hyperactivity	Primarily diagnostic/systemic biomarker	Seropositivity does not prove direct antibody-mediated ocular-surface injury	[29]
	Systemic lupus erythematosus	Dry eye, retinopathy, choroidopathy, optic neuropathy	ANA, anti-dsDNA, anti-Sm, antiphospholipid antibodies, complement	Diagnostic and mechanistically contributory	Different antibodies relate to different ocular phenotypes	[45,46]
	IgG4-related ophthalmic disease	Lacrimal-gland, extraocular-muscle, and orbital-nerve enlargement	Serum IgG4; tissue IgG4+ plasma cells; clonally expanded B cells	Diagnostic/pathobiological B-cell marker	Serum IgG4 alone is neither sensitive nor specific	[31]
**Boundary** **disorders:** **predominantly** **cellular or cytokine-mediated** **ocular inflammation**	Behçet uveitis	Relapsing nongranulomatous uveitis with retinal vasculitis; anterior, posterior, or panuveitis	No established disease-specific pathogenic ocular antibody	Boundary comparator: predominantly cytokine- and cell-mediated inflammation	Candidate antibodies have not been established as diagnostic or pathogenic markers	[47]
	Sarcoid uveitis	Uveitis, macular edema, epiretinal membrane, glaucoma	No established disease-specific pathogenic ocular antibody	Boundary comparator: granulomatous cellular inflammation	ACE and sIL-2R are supportive but neither disease-specific nor pathogenic antibodies	[48]
**B-cell malignancies**	Vitreoretinal lymphoma	Vitreous cells, sub-RPE infiltrates, chronic-uveitis mimic	IgH/IgK clonality, MYD88 L265P, IL-10 and IL-10/IL-6 ratio	Malignant B-cell/clonality biomarker	Integrate cytology, molecular tests, imaging and CNS evaluation	[49]
	Ocular adnexal lymphoma	Conjunctival, lacrimal, eyelid or orbital mass	Monoclonal B-cell population/immunoglobulin rearrangement	Malignant B-cell lineage marker	Subtype and infectious/autoimmune context affect interpretation	[50]

Abbreviations: CAR, cancer-associated retinopathy; MAR, melanoma-associated retinopathy; TRPM1, transient receptor potential cation channel subfamily M member 1; AQP4, aquaporin-4; IgG, immunoglobulin G; NMOSD, neuromyelitis optica spectrum disorder; MOG, myelin oligodendrocyte glycoprotein; ANCA, antineutrophil cytoplasmic antibody; PUK, peripheral ulcerative keratitis; PR3, proteinase 3; MPO, myeloperoxidase; GPA, granulomatosis with polyangiitis; SSA/Ro, Sjögren’s syndrome-related antigen A/Ro; SSB/La, Sjögren’s syndrome-related antigen B/La; ANA, antinuclear antibody; dsDNA, double-stranded DNA; anti-Sm, anti-Smith antibody; AChR, acetylcholine receptor; MuSK, muscle-specific kinase; MYD88, myeloid differentiation primary response 88; LRP4, low-density lipoprotein receptor-related protein 4; TSH, thyroid-stimulating hormone; ACE, angiotensin-converting enzyme; sIL-2R, soluble interleukin-2 receptor. RPE, retinal pigment epithelium; IgH, immunoglobulin heavy chain; IgK, immunoglobulin kappa light chain; CNS, central nervous system.

## Data Availability

No new data were created or analyzed in this study. Data sharing is not applicable to this article.
